# Supplementary material for: COVID-19 Vaccination Reduces Lower Limb Amputation Rates and Mortality Rate in Patients with Pre-Existing Peripheral Vascular Disease Based on TriNetX Database
Source: Vaccines (Basel). 2025 Sep 12;13(9):969. doi: 10.3390/vaccines13090969 (PMC12474159; doi:10.3390/vaccines13090969)
Supplement: Supplementary file 1 [file vaccines-13-00969-s001.zip › vaccines-3816806-supplementary.pdf]

Supplementary Table S1 Sensitivity analysis- risk of all-cause mortality by type of vaccine

Vaccine type	Event n	3-y prob.†	HR (95% CI)
BNT vs Unvaccinated			
BNT (n= 3,948)	655	18.5%	0.932(0.836-1.030)
Unvaccinated (n= 3,948)	652	19.1%	Reference
Moderna vs Unvaccinated			
Moderna (n= 1,717)	317	19.6%	0.924(0.790-1.000)
Unvaccinated (n=1,717)	312	20.8%	Reference
Adenoviral Vector vs Unvaccinated			
Adenoviral Vector (n= 97)	24	26.0%	1.436(0.771-2.670)
Unvaccinated (n= 97)	17	19.5%	Reference
Moderna vs BNT			
Moderna (n= 1,715)	316	19.6%	0.994(0.850-1.160)
BNT (n= 1,715)	310	19.9%	Reference

† 3-y prob., the 3-year cumulative probability was estimated by Kaplan-Meier method.

Supplementary Table S2 Sensitivity analysis- risk of ischemic stroke by type of vaccine

Vaccine type	Event n	3-y prob.†	HR (95% CI)
BNT vs Unvaccinated			
BNT (n= 3,948)	470	13.6%	1.105(0.967-1.260)
Unvaccinated (n= 3,948)	405	12.3%	Reference
Moderna vs Unvaccinated			
Moderna (n= 1,717)	192	12.6%	1.074(0.873-1.320)
Unvaccinated (n=1,717)	166	12.1%	Reference
Adenoviral Vector vs Unvaccinated			
Adenoviral Vector (n= 97)	<11	10.0%	0.716(0.288-1.780)
Unvaccinated (n= 97)	11	12.9%	Reference
Moderna vs BNT			
Moderna (n= 1,715)	191	12.6%	0.976(0.799-1.190)
BNT (n= 1,715)	192	13.0%	Reference

† 3-y prob., the 3-year cumulative probability was estimated by Kaplan-Meier method.

Supplementary Table S3 Sensitivity analysis- risk of lower limb amputation by type of vaccine

Vaccine type	Event n	3-y prob.†	HR (95% CI)
BNT vs Unvaccinated			
BNT (n= 3,948)	79	2.4%	0.783(0.580-1.050)
Unvaccinated (n= 3,948)	94	2.9%	Reference
Moderna vs Unvaccinated			
Moderna (n= 1,717)	31	2.3%	0.822(0.505-1.330)
Unvaccinated (n=1,717)	34	2.4%	Reference
Adenoviral Vector vs Unvaccinated			
Adenoviral Vector (n= 97)	<11	6.6%	1.617(0.386-6.770)
Unvaccinated (n= 97)	<11	4.6%	Reference
Moderna vs BNT			
Moderna (n= 1,715)	31	2.3%	0.967(0.588-1.590)
BNT (n= 1,715)	31	2.1%	Reference

† 3-y prob., the 3-year cumulative probability was estimated by Kaplan-Meier method.

Supplementary Table S4 Sensitivity analysis- risk of all-cause mortality by vaccine dose

Vaccine dose	Event n	3-y prob.†	HR (95% CI)
BNT 1 dose vs Unvaccinated			
BNT 1 dose (n= 178)	43	26.0%	1.144(0.732-1.780)
Unvaccinated (n=178)	35	21.5%	Reference
BNT 2 dose vs Unvaccinated			
BNT 2 dose (n= 1,086)	222	21.9%	1.126(0.926-1.300)
Unvaccinated (n= 1,086)	183	18.9%	Reference
BNT booster vs Unvaccinated			
BNT booster (n=1,964)	294	17.0%	0.777(0.666-0.900)
Unvaccinated (n=1,964)	355	20.7%	Reference
Moderna 1 dose vs Unvaccinated			
Moderna 1 dose (n=63)	17	27.6%	1.018(0.508-2.040)
Unvaccinated (n=63)	15	26.9%	Reference
Moderna 2 dose vs Unvaccinated			
Moderna 2 dose (n= 442)	111	25.8%	1.161(0.875-1.500)
Unvaccinated (n= 442)	85	22.3%	Reference
Moderna booster vs Unvaccinated			
Moderna booster (n=)	102	17.1%	0.867(0.660-1.130)
Unvaccinated (n=)	106	19.0%	Reference

† 3-y prob., the 3-year cumulative probability was estimated by Kaplan-Meier method.

1 dose refers to having received only one dose, with no record of a second dose or more.

2 doses refers to having received two doses, with no record of a third dose or a booster.

Supplementary Table S5 Sensitivity analysis- risk of ischemic stroke by vaccine dose

Vaccine dose	Event n	3-y prob.†	HR (95% CI)
BNT 1 dose vs Unvaccinated			
BNT 1 dose (n= 178)	21	13.8%	0.868(0.480-1.560)
Unvaccinated (n=178)	23	15.7%	Reference
BNT 2 dose vs Unvaccinated			
BNT 2 dose (n= 1,086)	145	15.2%	1.226(0.959-1.560)
Unvaccinated (n= 1,086)	113	12.5%	Reference
BNT booster vs Unvaccinated			
BNT booster (n=1,964)	231	13.4%	0.971(0.808-1.160)
Unvaccinated (n=1,964)	227	14.2%	Reference
Moderna 1 dose vs Unvaccinated			
Moderna 1 dose (n=63)	<11	10.9%	0.803(0.270-2.300)
Unvaccinated (n=63)	≤ 11	12.4%	Reference
Moderna 2 dose vs Unvaccinated			
Moderna 2 dose (n= 442)	47	11.9%	0.868(0.582-1.290)
Unvaccinated (n= 442)	49	13.1%	Reference
Moderna booster vs Unvaccinated			
Moderna booster (n=)	72	12.2%	1.208(0.852-1.710)
Unvaccinated (n=)	56	10.7%	Reference

† 3-y prob., the 3-year cumulative probability was estimated by Kaplan-Meier method.

1 dose refers to having received only one dose, with no record of a second dose or more.

2 doses refers to having received two doses, with no record of a third dose or a booster.

Supplementary Table S6 Sensitivity analysis- risk of lower limb amputation by vaccine dose

Vaccine dose	Event n	3-y prob.†	HR (95% CI)
BNT 1 dose vs Unvaccinated			
BNT 1 dose (n= 178)	<11	1.5%	0.155(0.035-0.690)
Unvaccinated (n=178)	12	8.5%	Reference
BNT 2 dose vs Unvaccinated			
BNT 2 dose (n= 1,086)	21	2.3%	0.625(0.359-1.080)
Unvaccinated (n= 1,086)	31	3.6%	Reference
BNT booster vs Unvaccinated			
BNT booster (n=1,964)	39	2.4%	1.021(0.649-1.600)
Unvaccinated (n=1,964)	36	2.5%	Reference
Moderna 1 dose vs Unvaccinated			
Moderna 1 dose (n=63)	0	0.0%	N.A.
Unvaccinated (n=63)	0	0.0%	Reference
Moderna 2 dose vs Unvaccinated			
Moderna 2 dose (n= 442)	<11	1.9%	0.443(0.179-1.090)
Unvaccinated (n= 442)	14	4.0%	Reference
Moderna booster vs Unvaccinated			
Moderna booster (n=643)	17	3.5%	1.181(0.573-2.430)
Unvaccinated (n=)	13	2.6%	Reference

† 3-y prob., the 3-year cumulative probability was estimated by Kaplan-Meier method.

1 dose refers to having received only one dose, with no record of a second dose or more.

2 doses refers to having received two doses, with no record of a third dose or a booster.

N.A., not available.
